# Parameter Estimation and Quantification of Magnetic Nanoparticles Based on Improved Particle Swarm Optimization

**DOI:** 10.3390/mi17010022

**Published:** 2025-12-25

**Authors:** Huangliang Wu, Hang Yu, Xiaoyu Chen, Yang Gao, Xiaolin Ning

**Affiliations:** 1 School of Instrumentation Science and Optoelectronic Engineering, Beihang University, Beijing 100191, China; wuhuangliang@buaa.edu.cn (H.W.); transover@buaa.edu.cn (H.Y.); xiaoyuchen03@buaa.edu.cn (X.C.); 2 Hangzhou Institute of National Extremely-Weak Magnetic Field Infrastructure, Hangzhou 310051, China; ningxiaolin@buaa.edu.cn; 3 Hefei National Laboratory, Gaoxin District, Hefei 230088, China

**Keywords:** MRX, magnetic nanoparticles, particle swarm optimization (PSO), parameter estimation and quantification

## Abstract

Magnetic Relaxometry (MRX) is a promising technique for probing the magnetic properties of nanoparticles with considerable potential in biomedical applications. It magnetizes magnetic nanoparticles through a direct current magnetic field to obtain measurable Néel relaxation signals when magnetic nanoparticles are combined with specific cells or antibodies. It employs highly sensitive magnetic sensors to record relaxation signals following nanoparticle magnetization, from which intrinsic parameters and quantitative information can be extracted, and ultimately completes mass detection. The essential step in MRX-based mass detection is to establish the calibration relationship between the relaxation signal amplitude reflecting the magnetic moment and the corresponding mass of magnetic nanoparticles. In this article, we present a parameter estimation and quantification framework that integrates an improved Particle Swarm Optimization (PSO) algorithm with the Moment Superposition Model (MSM) as the objective function. The proposed method effectively combines experimental data with a theoretical model, enabling accurate determination of key intrinsic parameters, including saturation magnetization and magnetic anisotropy. Building on these reliable estimating parameters, the proposed PSO algorithm is further applied to quantify nanoparticle mass. Validation through simulations and experimental data confirms the robustness of the method, with the final mass detection error reaching the microgram level. These results highlight its potential for precise characterization of magnetic nanoparticles in biomedical contexts.

## 1. Introduction

Magnetic nanoparticles (MNPs), as biocompatible magnetic enhancers, have attracted considerable interest for biomedical detection applications [1]. The relaxation principle of MNPs has been widely employed in cellular detection studies [2], where binding of MNPs to target cells induces characteristic changes in relaxation signals. These changes arise from the distinct contributions of Brownian and Néel relaxation [3,4]. Leveraging this signal specificity, high-sensitivity magnetic sensors can detect magnetic field variations to identify whether MNPs have successfully targeted specific cells, particularly cancer cells [4,5,6].

Among magnetization approaches, direct current (DC) magnetization followed by measurement of the subsequent relaxation process—referred to as magnetic relaxometry (MRX)—is the method adopted in this study. MRX offers clear advantages: (1) signal amplitudes are stronger than those of spontaneous biomagnetic signals (e.g., MEG or MCG), typically in the pT–nT range, with distinct temporal features that facilitate signal processing and modeling; (2) the signal source is controllable, as particle magnetic moments are fully aligned with the external magnetizing field, reducing the number of unknown parameters and minimizing propagation-related attenuation. Furthermore, owing to the transparency of biological tissues to magnetic fields, MRX achieves high signal-to-noise ratios in magnetic source imaging. However, quantitative detection using MRX typically requires experimental calibration across a series of samples, and different batches of nanoparticles must be recalibrated [5,7,8]. This article aims to develop a model-guided quantification approach to simplify the calibration process and reduce experimental complexity. Furthermore, due to the limited number of sensors, MRX exhibits relatively low spatial resolution in the biological applications of magnetic source imaging. To enhance resolution, the detection platform often needs to be repositioned, or additional magnetization coils need to be added to expand measurement coverage [9,10], which in turn further increases the measurement time.

Most current studies on MRX employ superconducting quantum interference devices (SQUIDs) as the signal detection and acquisition instruments [5,11], demonstrating the feasibility of SQUID systems for detecting MNPs as well as small numbers of cells. On this basis, small-coil magnetization schemes and hardware devices suitable for complex magnetic imaging have also been proposed [12,13]. Although SQUIDs offer high sensitivity and the ability to detect very small quantities of particles [12], they also suffer from high costs [14] and fixed layout constraints [8]. In recent years, researchers have proposed the use of optically pumped magnetometers (OPMs) as an alternative to SQUIDs for MRX measurements [15,16,17], and preliminary applications have already been demonstrated in biological models [18]. Moreover, appropriate nanoparticle preparation and selection are prerequisites for MRX signal detection. Among them, iron oxide-based MNPs are the most commonly used and exhibit relatively good biocompatibility [19]. The core size distribution of the particles is a key parameter to consider. Typically, particles suitable for MRX studies have core diameters in the range of approximately 23–30 nm [2,20]. According to relaxation theory, the particle size distribution and magnetocrystalline anisotropy affect the relaxation time of the particles, while the particle saturation magnetization and particle quantity influence the relaxation signal intensity. For tumor detection using MRX, the particles only need to exhibit clearly distinguishable Néel and Brownian relaxation characteristics.

Theoretical modeling of MRX has often relied on the moment superposition model (MSM), first introduced by Chantrell et al. [21], which conceptualizes an ensemble of MNPs as a non-interacting collection of magnetic moments, with total magnetization represented as the linear sum of all moments. It has been widely applied to interpret nanoparticle relaxation processes [22,23,24,25], and studies such as Adolphi et al. [26] have demonstrated that anisotropy parameters critically influence simulation outcomes, highlighting uncertainties in estimating intrinsic parameters. The MSM has been widely employed to explain the magnetic relaxation of MNPs and can provide prior information regarding particle properties [22,23,24,25]. Nevertheless, current studies largely rely on empirical models derived from experimental data. Flynn et al. [5] developed a multi-parameter distributed fitting method, Huang et al. [27] proposed a more comprehensive fitting model applicable to different experimental conditions, and Stefan et al. [28] introduced an automated preprocessing method for SQUID-MRX data. However, these approaches are fundamentally based on experimental fitting and lack guidance from the MSM, particularly regarding the influence of key model parameters on experimental data. Therefore, effectively integrating a theoretical model with experimental data in parameter estimation and quantification remains a critical challenge in MRX studies. In particular, developing a methodology that is guided by a theoretical model and incorporates experimental data for parameter estimation is of considerable significance, enabling more accurate mass estimation based on precisely estimated parameters.

This article aims to develop a framework based on an improved PSO algorithm to achieve quantitative analysis of magnetic nanoparticles under unknown intrinsic parameters. The primary objectives include (1) extracting intrinsic particle parameters and particle mass from Néel relaxation signals and (2) integrating the MSM effectively to obtain more accurate estimations.

## 2. Materials and Methods

This article proposes a novel framework for parameter estimation and a quantification method of magnetic nanoparticles (MNPs) based on an improved Particle Swarm Optimization (PSO) algorithm. Section 2.1 outlines the methodology. First, the theoretical model of nanoparticle relaxation signals and the parameters related to nanoparticle morphology and magnetic properties are introduced. Next, an improved PSO algorithm, a mathematical optimization technique, is described, along with two iterative enhancement strategies designed to overcome convergence stagnation. By utilizing the MSM, a unified framework for parameter estimation and quantification is established, and the processes of estimation and quantification are conducted in a staged manner. Section 2.2 describes the experimental setup and the magnetic properties of the MNPs. The overall experimental layout is first presented, including the use of an optically pumped magnetometer (OPM) for signal detection and the magnetization control device. The testing procedures, as well as signal acquisition and processing methods, are then detailed. Finally, the nanoparticle size distribution and magnetic properties of MNPs employed in this article are introduced.

### 2.1. Relaxation Theory Models and Parameter Estimation Methods

#### 2.1.1. Relaxation Mechanisms of Magnetic Nanoparticles

The relaxation of magnetic nanoparticles (MNPs) can be induced after direct current (DC) magnetization [5,15,17]. Brownian relaxation is mainly observed when the MNPs remain freely movable, whereas Néel relaxation occurs when they are immobilized. Given that MNPs are immobilized after binding to tumors, the relaxation characterization in this article is limited to Néel relaxation due to this application. The DC relaxation mode of MNPs follows an exponential decay, in which two characteristic parameters are of primary interest: the initial amplitude and relaxation time. The Néel relaxation time of MNPs is determined by their intrinsic properties. In general, the Néel relaxation time of MNPs can be expressed as [5]

(1) $$\tau_{N}=\tau_{0}\exp\frac{KV_{p}}{k_{B}T},$$

where $\tau_{0}$ denotes the characteristic relaxation time, *K* is the magnetic anisotropy of the MNPs, $V_{p}$ represents the volume of the magnetic core, $k_{B}$ is the Boltzmann constant, and *T* is the absolute temperature in Kelvin. The initial amplitude of the relaxation signal generated by MNPs is determined by their intrinsic properties as well as the magnetization conditions, which are reflected in the magnetic relaxation data simulation model described in Section 2.1.2. The decay-related characteristic parameters of MNPs are subsequently employed as the cost function for quantitative analysis and parameter estimation in Section 2.1.3.

#### 2.1.2. Magnetic Relaxation Data Simulation Model

The simulation model for magnetic nanoparticle relaxation is based on the Moment Superposition Model (MSM) [22,23,24,25]. The magnetization and relaxation processes are calculated separately for a system of MNPs with a given nanoparticle size distribution. For the convenience of research, interparticle interactions are assumed to be negligible prior to computation. This assumption is consistent with the actual relaxation behavior and significantly simplifies the MSM. First, the magnetization process of the MNPs is modeled. The magnetization of MNPs can be described by the Langevin function as follows:

(2) $$M_{H}=M_{S}L\left(\frac{\mu_{0}HM_{S}V_{p}}{k_{B}T}\right),$$

here, *M_H_* denotes the net magnetization of the MNPs after magnetization, *M_S_* is the saturation magnetization, *H* represents the applied magnetic field strength, and $\mu_{0}=4\pi \times 10^{-7}\;N/A^{2}$ is the permeability of the free space. The Langevin function, $L\left(\cdot \right)$, characterizes the magnetization process of the MNPs.

The proportion of the magnetic nanoparticle system that undergoes Néel relaxation, *N_p_*, can be calculated based on the magnetization time and is expressed as follows [2,29]:

(3) $$N_{p}=1-\exp\frac{t_{mag}}{\tau_{N,mag}},$$

here, *t_mag_* denotes the magnetization time, and $\tau_{N,mag}=\tau_{N}\left(1-0.82\mu_{0}HM_{s}/K\right)$ represents the relaxation time under the applied magnetic field *H*. Therefore, the magnetization ratio is determined by the relaxation time and the magnetization time. It is derived based on the potential energy change when the MNPs transform from the susceptible axis direction to the direction of the applied magnetic field under the applied magnetic field [30]. Eberbeck et al. [22] simplified the quadratic term in potential energy change, solved for the expression with only the linear term retained, and calculated the theoretical coefficient of the linear term as 0.82.

Based on the magnetized proportion, the relaxation function can be expressed as the decay of the magnetized magnetic moment, also in an exponential form. Assuming that the system contains *n* MNPs that undergo Néel relaxation, the Néel relaxation magnetic moment of the nanoparticle system can be expressed as follows [26]:

(4) $$u\left(t\right)=nN_{p}M_{H}V_{p}\exp\left(-\frac{t}{\tau_{N}}\right),$$

here, *t* represents a single time point or the time axis following Néel relaxation.

In fact, the magnetic core volumes of MNPs exhibit a certain distribution rather than a single fixed value. Therefore, other measurement techniques such as electron microscopy (EM) or dynamic light scattering (DLS) are required to determine the volume distribution of the nanoparticle system [31]. All MNPs are approximated as spheres, and the volume distribution is equivalently modeled using a log-normal probability density function for nanoparticle size [30], expressed as follows:

(5) $$f\left(d_{p}\right)=\frac{1}{d_{p}\sigma \sqrt{2\pi }}\exp\left(-\frac{\ln^{2}\left(d_{p}/m\right)}{2\sigma^{2}}\right),$$

here, *d_p_* is the magnetic core diameter corresponding to the magnetic core volume *V_p_*, σ denotes the nanoparticle size standard deviation, and *m* represents the mean nanoparticle diameter. From this, the nanoparticle volume distribution log-normal probability density function *f(V_p_)* corresponding to the nanoparticle diameter distribution log-normal probability density function *f(d_p_)* can be obtained.

Accordingly, the overall Néel relaxation of the nanoparticle system can be expressed as a modification of Equation (4) in Equation (5):

(6) $$u\left(t\right)=\int nN_{p}M_{H}f\left(V_{p}\right)\exp\left(-\frac{t}{\tau_{N}}\right)dV_{p},$$

here, during the transformations between *V_p_* and *d_p_*, as well as the conversions of *f(d_p_)* and *f(V_p_)* and their respective integrals, it is necessary to unify the integration variable and adjust the integration limits accordingly. The number of MNPs can be calculated based on the volume of the nanoparticle system, expressed as follows:

(7) $$n=\frac{m/\rho_{nano}}{\int V_{p}f\left(V_{p}\right)dV_{p}},$$

here, *ρ_nano_* is the density of the magnetic nanoparticle material, and *m* represents the mass of the nanoparticle system used. For simplicity, the mass of MNPs within the nanoparticle system (the colloidal state) is adopted as the quantification result.

The MSM calculates the magnetic moment of the nanoparticle system. To compare and validate with the experimentally measured magnetic field signal obtained by the sensor, a distance parameter must be introduced to convert the magnetic moment into the relaxation magnetic field signal. The sensor is positioned directly above the MNPs. According to the Biot–Savart law, the relationship between magnetic field signal and magnetic moment can be simplified as a proportional coefficient related to the distance, expressed as follows:

(8) $$b\left(t\right)=\frac{\mu_{0}}{4\pi }\cdot \frac{2}{d}\cdot u\left(t\right),$$

here, *b(t)* represents the converted magnetic field signal strength, and *d* denotes the effective distance between the nanoparticle system and the sensor.

#### 2.1.3. Improved Particle Swarm Optimization Algorithm

The relaxation signal of MNPs provides information characterizing their state. In the relaxation model described above, the signal is determined by both the intrinsic parameters of the MNPs and the magnetization conditions. The mean and standard deviation of the nanoparticle size are easily measured and thus can be regarded as known intrinsic parameters. Accordingly, the other intrinsic parameters governing the relaxation signal are the saturation magnetization *M_S_* and the magnetic anisotropy *K* of MNPs. While *M_S_* can be obtained from the VSM hysteresis loop, *K* is difficult to determine experimentally and varies significantly among different materials. Moreover, the nanoparticle mass directly determines the relaxation signal intensity.

In this article, a quantitative analysis method based on the MSM is proposed to perform effective parameter and quantitative estimation even when the intrinsic parameters as prior knowledge are insufficient. Under DC magnetization, the relaxation signal exhibits a pronounced exponential decay over time, which can determine intrinsic parameters: the saturation magnetization *M_S_* and magnetic anisotropy *K*, which is associated with the temporal characteristics of the signal, particularly the Néel relaxation time. Once these parameters are determined or equivalently determined, the only remaining unknown is the nanoparticle mass, which can be calculated from the initial relaxation signal strength. Due to the nonlinearity of the MSM and the differences in magnitude of its outputs, the PSO algorithm demonstrates significant advantages for both parameter and mass estimation.

The PSO algorithm is a heuristic optimization method first proposed by Eberhart and Kennedy in 1995 [32]. Its core principle is to simulate the foraging behavior of bird flocks, searching for the optimal solution through information sharing at both the population and individual levels [33]. In this article, the saturation magnetization *M_S_*, magnetic anisotropy *K*, and nanoparticle mass are used as particle features in the PSO algorithm. An *M*-dimensional space is established, where the position of the *i*-th particle in the space is represented as follows:

(9) $$X_{i}=\left[x_{im}\right]=\left[x_{i1},x_{i2},\ldots ,x_{iM}\right],$$

here, $x_{im}={\left[K\;M_{s}\;V\right]}^{T}$.

The particles are manipulated as follows:

(10) $$\begin{array}{cc} x_{im}\left(k\right) & =x_{im}\left(k-1\right)+v_{im}\left(k\right)\\ v_{im}\left(k\right) & =\omega \cdot v_{im}\left(k-1\right)\\ & +c_{1}\cdot r_{1}\cdot \left(p_{im}-x_{im}\left(k-1\right)\right)\\ & +c_{2}\cdot r_{2}\cdot \left(p_{gm}-x_{im}\left(k-1\right)\right) \end{array},$$

here, *v_im_* denotes the updated velocity, ω is the inertia weight, and *c_1_* represents the cognitive learning factor. *r_1_* is a random number matrix uniformly distributed in the interval [0, 1] with dimensions matching the space. *p_im_* denotes the historical best position of particle *i* in the *m*-th dimension at the *k*-th iteration. Similarly, *c_2_* is the social learning factor, *r_2_* is another random number matrix uniformly distributed in the interval [0, 1], and *p_gm_* denotes the global best position in the *m*-th dimension at the *k*-th iteration.

**Figure 1:**
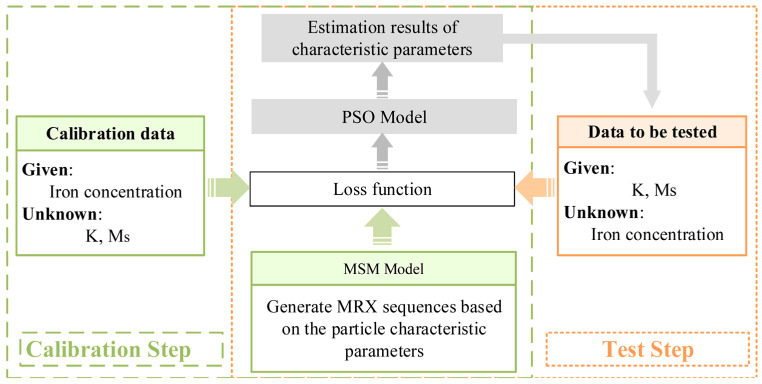
The parameter estimation and quantification method framework of the Particle Swarm Optimization algorithm combined with the MSM.

Based on the MSM, a parameter estimation and quantitative analysis method using the PSO algorithm is proposed when prior information is unknown. When the intrinsic magnetic parameters of MNPs, the saturation magnetization *M_S_* and magnetic anisotropy *K*, are either both unknown or only one is known, the relaxation signal under a known nanoparticle mass is first employed for estimation of *M_S_* and *K*. The estimated parameters are then treated as known information, and quantitative estimation is subsequently performed using the same PSO algorithm. The overall estimation workflow is illustrated in Figure 1.

The optimization objective function $F_{nano,MSM}$ is designed based on the MSM and the relaxation signal and is defined as follows:

(11) $$\begin{array}{cc} F_{nano,MSM} & =abs\left(u_{nano}\left(0\right)-u_{{\mathrm{MSM}}_{k}}\left(0\right)\right)\\ & +abs\left(t\_mrx_{nano}-t\_mrx_{{\mathrm{MSM}}_{k}}\right) \end{array},$$

here, $u_{nano}\left(0\right)$ denotes the initial value of the simulated or measured relaxation signal generated by MNPs, while $u_{{\mathrm{MSM}}_{k}}\left(0\right)$ represents the theoretical initial value of the relaxation signal calculated by the MSM using parameter $x_{im}$ at the *k*-th iteration. $t\_mrx_{nano}$ denotes the Néel relaxation time of the relaxation signal, and $t\_mrx_{{\mathrm{MSM}}_{k}}$ represents the theoretical Néel relaxation time calculated by the MSM using parameter $x_{im}$ at the *k*-th iteration.

**Figure 2:**
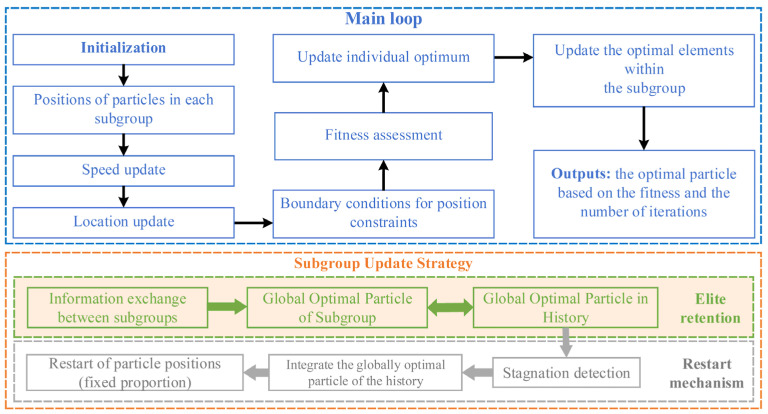
Flowchart of the improvement strategy for the Particle Swarm Optimization algorithm.

According to the modeling method of Néel relaxation signal described in Section 2.1.2, the objective function resulting from different parameter combinations is discontinuous and exhibits non-smooth gradients. In fact, parameter estimation of MNPs constitutes a typical non-convex optimization problem, which will be further discussed in the Discussion section. Based on this, an improved strategy for the traditional PSO algorithm is proposed, as illustrated in Figure 2. The main improvements are summarized as follows:

1. Multiple Subgroups: Several subgroups are formed, with each subgroup independently performing particle updates within the main PSO loop. The particle update process is conducted under boundary constraints, producing both the individual best positions for each particle and the global best position of the subgroup;
2. Elite preservation mechanism: During the iterations, subgroups exchange information to determine the global best particle across all subgroups. The first updated global best particle is used as the initial historical global best particle. In subsequent iterations, the global best particle obtained in the current iteration is compared with that of the previous iteration. If the current global best particle yields a smaller objective function value, it replaces the previous one as the new historical global best particle. A predefined number of historical global best particles is maintained, and at each iteration, no more than this number of particles can be updated;
3. Stagnation-restart mechanism: A maximum stagnation iteration threshold is first defined. If the historical global best particle is not updated within this number of iterations, a fixed proportion of particles in each subgroup is re-initialized. The restart procedure ensures that the current historical global best particle is preserved within each subgroup while maintaining inter-subgroup information exchange. A ring topology is adopted for inter-subgroup communication, where subgroups are arranged sequentially, and the best particle from a neighboring subgroup is used to influence the particles of the current subgroup through mutation. In this article, the mutation probability in the subgroup information exchange is set to a random value not exceeding 20%. Specifically, a mutation vector is added to the position of each subgroup’s global best particle, which is calculated as the product of a random probability and the difference between the global best particles of adjacent subgroups. This mechanism effectively mitigates stagnation by introducing controlled diversity while retaining superior solutions.

### 2.2. Experiment Setup

**Figure 3:**
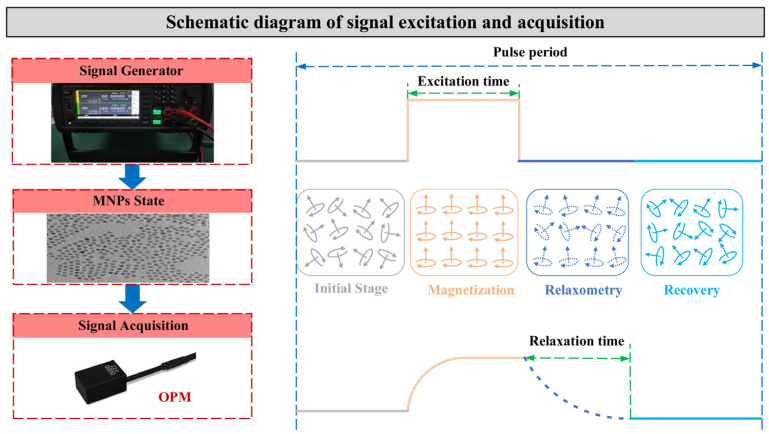
Schematic diagram of magnetic relaxation experiment.

In this article, magnetic iron oxide nanoparticles were employed as magnetic relaxation detection agents. The nanoparticle size is defined as the core diameter, excluding the surface coating layer, because only the iron oxide core possesses magnetism. For convenience of modeling and analysis, the single-core or equivalent single-core MNPs were utilized in this article. The generation and acquisition of the relaxation signal are illustrated in Figure 3. In a single trial, the Optically Pumped Magnetometer (OPM) collects the complete temporal signal of the nanoparticle system after magnetization by the excitation device.

#### 2.2.1. Measurement by OPM

**Figure 4:**
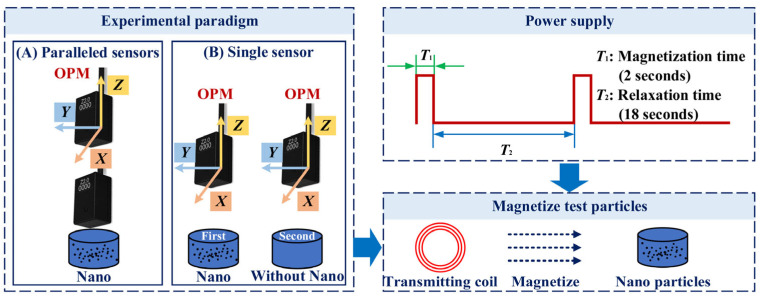
Schematic diagram of the experimental paradigm and test steps.

The second-generation Quspin sensor (an OPM device manufactured by Quspin Company, Louisville, CO, USA) was employed in this article to detect the Néel relaxation signals of MNPs. A gradient measurement configuration was adopted, as illustrated in Figure 4A, in which the two sensors, the center of the nanoparticle sample, and the coil axis were aligned along the same straight line. In the single-sensor configuration, shown in Figure 4B, background signals without MNPs were recorded and subsequently subtracted to obtain the gradient. The MNPs were placed in a cylindrical container with a base radius of 2.5 mm, whose base center was located at the center of the testing platform. The platform center was defined as the coordinate origin, and the sensitive axis of the sensor was aligned with the *z*-axis, perpendicular to the platform. In the single-sensor configuration, the sensor position was set to [0, 0, 0.02] (m), while in the gradiometer configuration, the two sensors were placed at [0, 0, 0.02] (m) and [0, 0, 0.04] (m), respectively.

#### 2.2.2. Magnetization Devices and Control Equipment

Magnetization of the MNPs was performed using a one-dimensional Helmholtz coil with the following structural parameters: radius of 2.5 cm, 180 turns, and a coil constant of 0.077 mV/mT. A programmable constant-voltage source provided controllable pulse currents to generate the pulsed magnetic field. In the pulse sequence, the magnetization phase (T1 in Figure 4) generated a magnetic field of several mT (tens of Gauss), while the relaxation phase (T2 in Figure 4) ensured that the magnetic field noise was at the pT level or below. The magnetization direction was aligned with the *z*-axis. In addition to the controllable power supply, a current-limiting module was integrated into the control system to ensure that leakage currents during the relaxation phase did not introduce magnetic noise that could interfere with the detection of the nanoparticle relaxation signal.

#### 2.2.3. Signal Acquisition and Processing

The host computer simultaneously acquired the pulse sequence output from the constant-voltage source and the full signal output from the OPM. The relaxation portion of the signal, which corresponds to the Néel relaxation of the MNPs, was extracted from the full OPM signal using the pulse sequence as a reference. A sliding filter was applied to remove 50 Hz electrical noise.

#### 2.2.4. Characterization of Magnetic Nanoparticles

The MNPs used in this article are PEG-coated Fe_3_O_4_ magnetite nanoparticles. The colloidal density of the magnetic nanoparticles is nominally 5 mg/mL. The MNPs were prepared via a high-temperature thermal decomposition method [34] and are nearly spherical in shape, with particle sizes primarily around 40 nm, and exist as colloidally hydrated nanoparticles. To ensure uniformity in the nanoparticle density during each sample collection for testing, ultrasonic vibration is applied prior to sampling to ensure the consistency of the nanoparticle density within the colloid.

**Figure 5:**
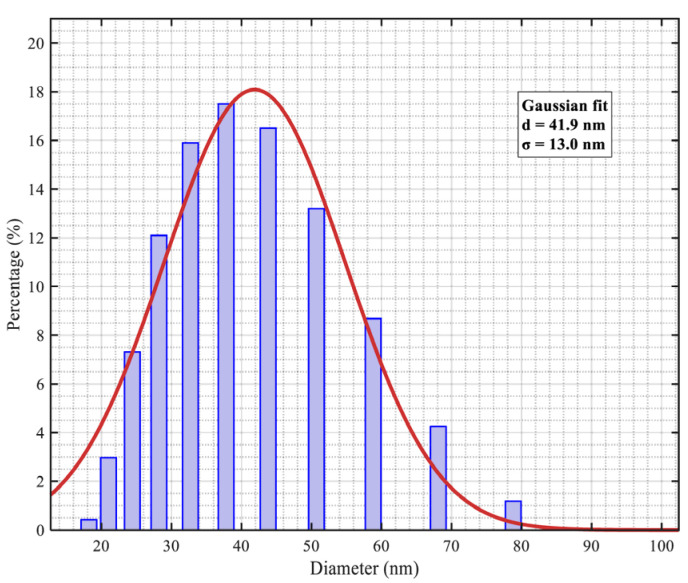
The particle size distribution count of MNPs and the logarithmic fitting curve graph.

Transmission Electron Microscopy (TEM) was used to characterize the MNPs. A 50 μL (50 μg MNPs) sample of colloid was used, and ten regions with uniform dispersion and a clean background were selected for imaging. The nanoparticle sizes were then divided into ten intervals, and the number of MNPs within each interval was counted. Then a log-normal distribution was applied to fit the size distribution data. The results are shown in Figure 5. The calculated average particle diameter *d_p_* of the nanoparticle system is 41.9 nm, with a nanoparticle size standard deviation σ of 13.0 nm.

**Figure 6:**
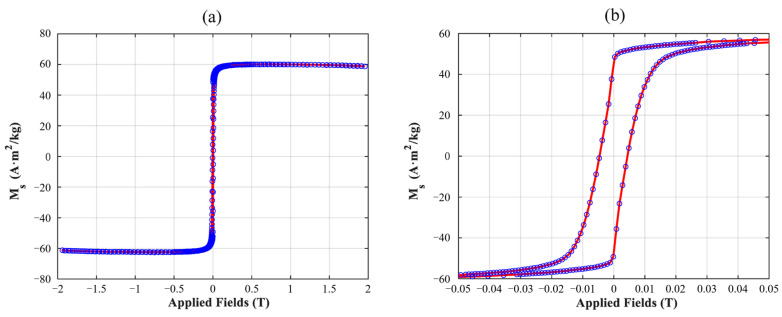
Magnetic hysteresis loop of MNPs under (**a**) a high magnetic field and (**b**) a low magnetic field. The blue circles depict the discrete test data points, and the red curve represents the corresponding fitting results.

In addition, the hysteresis loop of the nanoparticle system was measured using a Vibrating Sample Magnetometer (VSM) [35]. As shown in Figure 6a, saturation magnetization *M_S_* of the MNPs is obtained as 60.85 Am^2^/kg on average. For MNPs below 100 nm, the magnetic nanoparticles employed in this article exhibit a relatively high and authentic saturation magnetization compared with the commonly used SHP-series samples in previous studies [26], whose saturation magnetization values for SHP-20, SHP-25, SHP-30, and SHP-35 are 67.3, 85.0, 51.1, and 67.9 Am^2^/kg, respectively (with the numerical label indicating the mean nanoparticle size). In addition, as shown in Figure 6b, the MNPs display a remanence of approximately 48 Am^2^/kg and a coercivity of about 44 Oe. The estimated value of the saturation magnetization in this article can be compared and validated against the results from the measured hysteresis loop.

## 3. Results

Based on the approach mentioned above using the improved PSO algorithm, the effectiveness of the method was verified through simulation. Then, parameters and quantitative estimations were conducted using measured data, and finally, the estimation errors were analyzed. Relative error, defined as the ratio of the difference between the estimated value and the true value to the true value, is used for error analysis of both parameters and mass in the simulation and experimental procedures. In the simulation, the true values of the parameters and mass are predetermined and therefore exact. For the experimental data, the true saturation magnetization is taken as the average value obtained from VSM results; since magnetic anisotropy is difficult to measure directly, only the consistency of its estimated results is evaluated. In addition, the mass of each experimental sample is accurately known, enabling the calculation of the final relative error and error magnitude.

### 3.1. Simulation Validation

According to the MSM, relaxation simulation signals were generated for parameter estimation, with the signal generation method following Equation (6). First, several representative relaxation characteristic parameters were selected, and the magnetization parameters corresponding to the experimental conditions were set to generate the simulated Néel relaxation signals of MNPs. By combining different intrinsic parameters, namely saturation magnetization *M_S_* and magnetic anisotropy constant *K*, a set of simulated relaxation signals was established. To assess the effectiveness of the proposed method in distinguishing nanoparticle characteristics and performing quantitative detection, multiple simulation groups were generated under identical magnetization conditions but with varying nanoparticle parameters.

Since the number of MNPs in the system typically reaches the order of 10 to the power of several tens, it was converted to the mass of MNPs using Equation (7). Four combinations of nanoparticle mass and intrinsic parameters were selected to generate simulated Néel relaxation signals using the MSM. The specific parameter configurations are listed in Table 1. For each simulated signal, both parameter and mass were estimated using the improved PSO algorithm described in Section 2.1.3.

**Figure 7:**
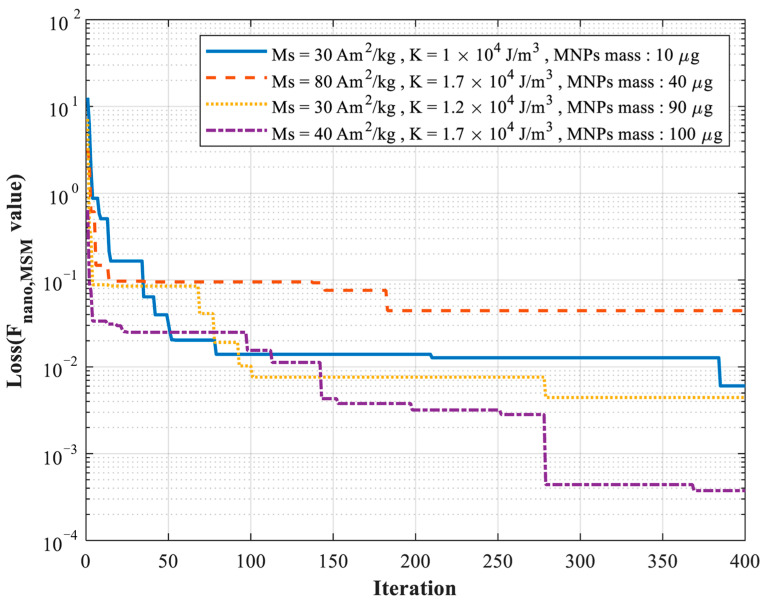
Schematic diagram of the iterative curve of parameter estimation for simulation data based on the improved PSO algorithm.

Figure 7 illustrates the iterative decay of the loss function during the optimization process for different sets of simulated relaxation signals. It can be observed that the improved PSO algorithm effectively avoids stagnation in the search for optimal parameters, and the optimal solutions can be obtained within approximately 400 iterations, demonstrating both convergence efficiency and robustness of the proposed method. Furthermore, the results across different simulated signal sets indicate that the algorithm exhibits strong adaptability to varying initial conditions and intrinsic parameter combinations, reliably approaching near-global optimal solutions under diverse scenarios. These findings further validate the applicability and effectiveness of the improved PSO algorithm for parameter estimation of MNPs.

**Figure 8:**
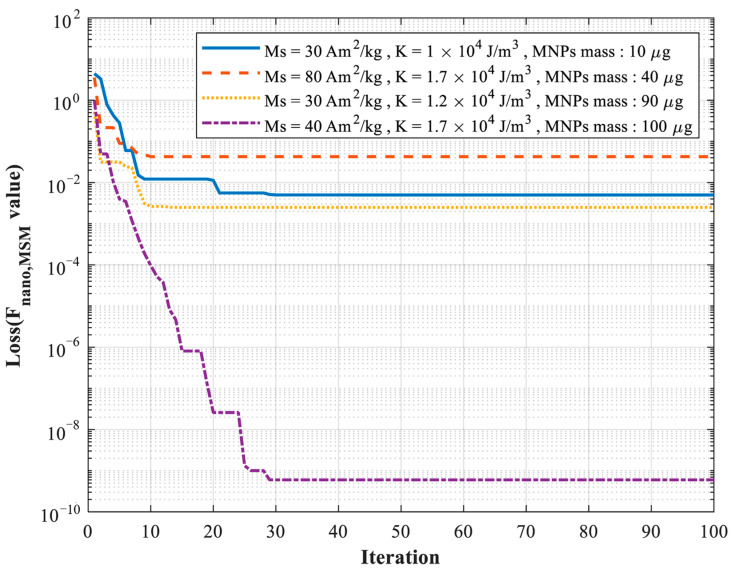
Schematic diagram of the iterative curve for mass estimation of simulation data based on the improved PSO algorithm.

Figure 8 presents the loss function decay process during mass estimation using the improved PSO algorithm under the condition that the saturation magnetization *M_S_* and magnetic anisotropy constant *K* were predetermined using the same algorithm. Because only a single parameter, the mass of the MNPs, was required to be estimated in this step, the optimization process exhibited significantly faster convergence compared to the estimation of *M_S_* and *K*. As shown in Figure 8, the optimal solution was reached within approximately 40 iterations, indicating that the proposed method can accurately and efficiently estimate the mass of the MNPs once the intrinsic magnetic parameters, *M_S_* and *K*, are determined.

The results of the parameter and mass estimation under four different combinations of intrinsic parameters and mass are shown in Table 1. In all cases, the intrinsic parameters and mass estimation errors are all within 1%, demonstrating the robustness and high accuracy of the proposed method. These results confirm that the improved PSO algorithm can effectively estimate both the magnetic parameters related to Néel relaxation and the mass of MNPs, thereby validating the applicability of the algorithm to the MSM-based parameter estimation problem. It leads to an important conclusion: combining Néel relaxation signals with the theoretical model (MSM) enables more accurate determination of both intrinsic parameters and mass. To further assess the practical performance of the proposed method, experimental data are introduced in the following section for parameter and mass estimation.

### 3.2. Analysis of Measured Data

**Figure 9:**
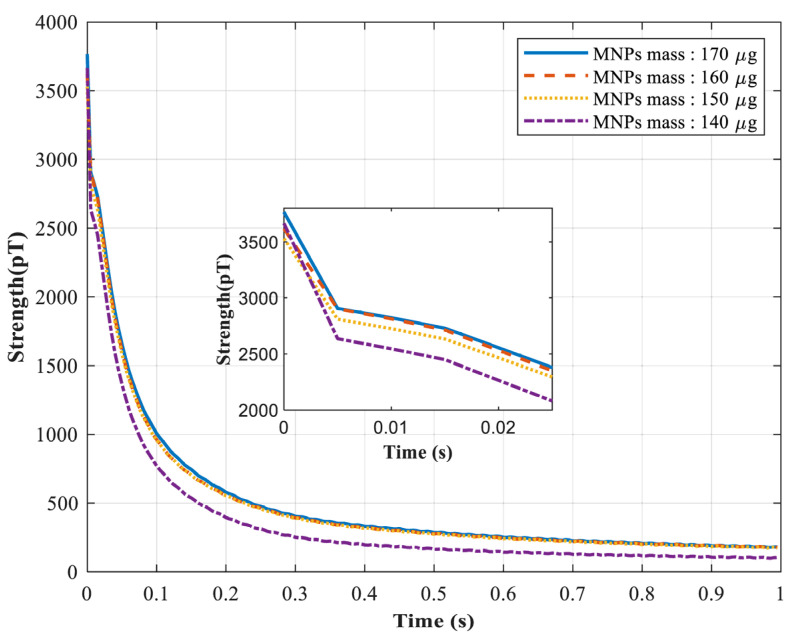
Schematic diagram of the measured Neel relaxation signals after processing under four different masses of MNPs.

Following the measurement and data processing described in Section 2.2, the parameter and mass estimation was conducted using experimental data with a controlled mass gradient. Figure 9 presents the processed Néel relaxation decay signals under four different mass conditions, where the first 1 s of signals are shown and the first 25 ms are highlighted. Based on these experimental signals, the proposed method was applied to estimate the intrinsic magnetic parameters, namely the saturation magnetization *M_S_* and magnetic anisotropy constant *K,* as well as to quantify the mass of MNPs.

**Figure 10:**
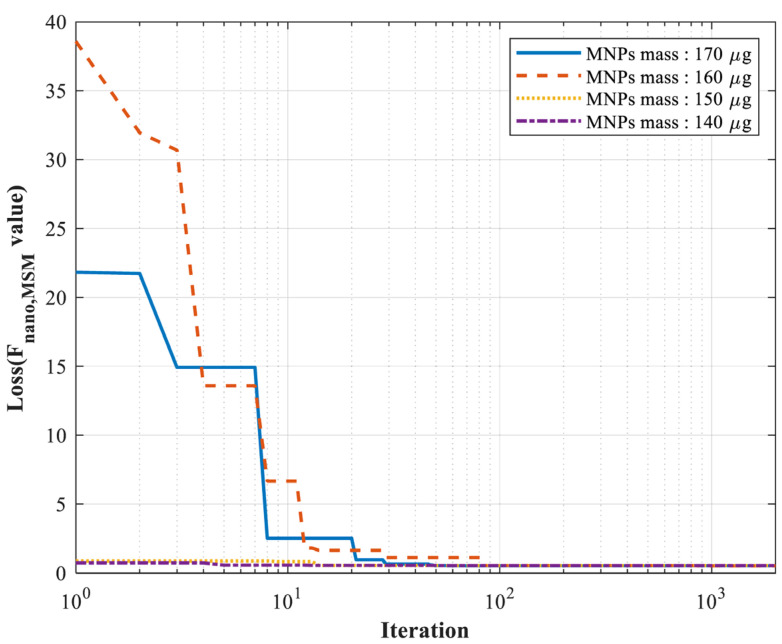
Schematic diagram of the iterative curve of parameter estimation based on the improved PSO algorithm for measured data.

To obtain parameter estimates consistent with the MSM and closely reflect experimental conditions, 2000 iterations were performed during the parameter estimation process to ensure that the improved PSO algorithm minimized the loss function. The convergence trajectories, illustrated in Figure 10, indicate that although the iteration curves depend on the choice of initial values, sufficiently large iteration numbers guarantee convergence to optimal solutions. These observations further highlight the robust convergence and stability of the improved PSO algorithm when applied to the nonlinear MSM with parameters spanning different magnitudes.

Table 2 summarizes the estimated intrinsic parameters under different mass conditions. The saturation magnetization *M_S_* was consistently estimated at approximately 60 Am^2^/kg, while the magnetic anisotropy constant *K* remained near 1.6 × 10^4^ J/m^3^. The estimated value of *M_S_* is consistent with the VSM results mentioned in Section 2.2.4, with an error within 4%. These results confirm the consistency of intrinsic magnetic properties for the same type of MNPs, thereby validating the effectiveness of the proposed method. Furthermore, using the estimated *M_S_* and *K* values, the mass of MNPs was estimated through the same improved PSO algorithm. The results in Table 2 demonstrate that, given intrinsic parameter estimation accuracy within 5%, the error in mass estimation is maintained at the microgram (μg) scale. Overall, these findings establish the applicability of the proposed approach under experimental conditions, enabling both reliable magnetic parameter estimation and accurate nanoparticle quantification.

## 4. Discussion

Magnetic nanoparticles generate relaxation signals upon immobilization, for example, when they bind to cells or antibodies. The mass of MNPs serves as a key indicator of the cell number they are bound to, making accurate quantitative estimation essential for their biomedical applications. The MSM-based relaxation model provides a theoretical framework to support and validate experimental results, while it can also guide data processing strategies based on measured relaxation signals. Within this framework, the accuracy of prior parameter (*M_S_* and *K* in this article) estimation directly determines the reliability of nanoparticle quantification.

**Figure 11:**
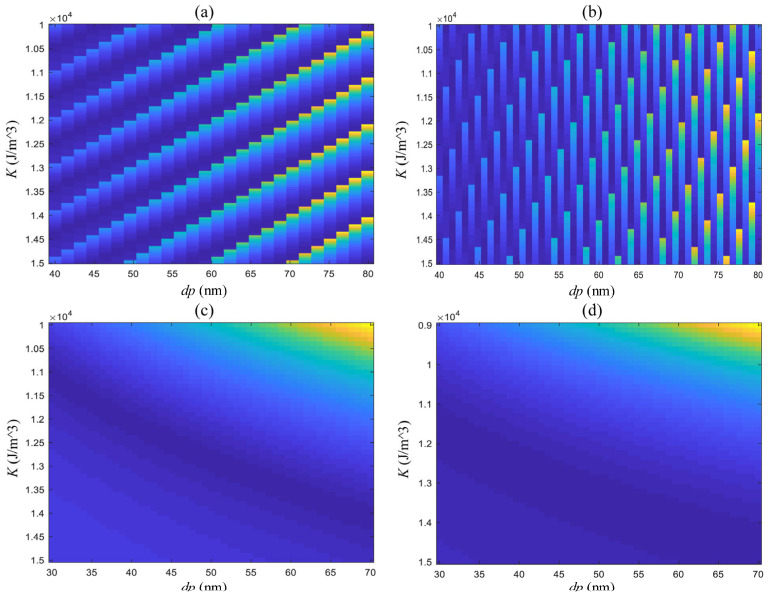
Schematic diagram of the distribution of objective functions of saturation magnetization and magnetic anisotropy under different step lengths: (**a**) *K*: 100 J/m^3^, $M_{S}$ : 0.1 Am^2^/kg; (**b**) *K*: 200 J/m^3^, $M_{S}$: 0.3 Am^2^/kg; (**c**) *K*: 100 J/m^3^, $M_{S}$: 0.8 Am^2^/kg; (**d**) *K*: 120 J/m^3^, $M_{S}$: 0.8 Am^2^/kg.

In this article, an improved PSO algorithm is introduced to perform both parameter estimation and quantitative analysis. The method lies in achieving an accurate estimation of intrinsic magnetic parameters—saturation magnetization *M_S_* and magnetic anisotropy *K*—under conditions where these parameters are initially unknown. As shown in Figure 11a,b, the objective function values under different parameter combinations indicate that finding the optimal parameter combination is a typical non-convex optimization problem. Only under specific parameter ranges and fixed step sizes can the parameter combination be transformed into a convex optimization problem, as shown in Figure 11c,d. However, during the optimization process, a fixed step size can lead to problems with convergence speed and optimization. In addition, if the step size were set in every iteration based on the estimated range, allowing it to be transformed into a convex optimization problem, the computational demands would be greatly amplified. Furthermore, within a larger parameter estimation range, there may not exist a step size that can be utilized to transform the problem into a convex optimization problem.

To address the existing non-convex optimization problem, we propose a subgroup-based PSO strategy incorporating elite preservation and stagnation-restart mechanisms to avoid becoming trapped in too many local optimal values. This method ensures robust convergence toward global optima in the estimation of *M_S_* and *K*, thereby enabling accurate determination of nanoparticle mass through the same optimization framework.

In summary, the proposed method integrates theoretical modeling with experimental data to enable more accurate estimation of intrinsic parameters and nanoparticle mass. This approach can be directly applied to quantitative studies of MNPs and further serves as a validation framework for alternative data-processing strategies and spatial distribution reconstruction methods based on relaxation signals for other scholars.

## 5. Conclusions

This article presents a two-stage framework for parameter estimation and quantitative analysis of Néel relaxation signals from MNPs. Through designing an objective function based on the MSM and employing an improved PSO algorithm, the proposed method can estimate the intrinsic parameter and mass of MNPs in sequence. The simulation results indicate that the improved PSO algorithm can accurately perform parameter estimation and mass detection, addressing the non-convex optimization issue in the objective function. Both parameter and mass errors are within 1%. Experimental data results demonstrate that the proposed method is applicable under actual measurement conditions, effectively estimating the characteristic parameters of the magnetic nanoparticles with an error of approximately 5%. The average detection error for mass is two orders of magnitude smaller than the total mass, reaching the microgram (µg) level, with a maximum error of 0.52 µg. The combination of the MSM and experimental data presented in this study provides a quantitative detection approach, which, especially in the case of unknown saturation magnetization and magnetic crystalline anisotropy, is expected to contribute to the advancement of research on the quantitative detection of magnetic nanoparticles.

## Figures and Tables

**Table 1:** Estimation results of parameters and iron mass under simulation conditions.

	Trial 1	Trial 2	Trial 3	Trial 4
Real *K* (×10^4^ J/m^3^)	1	1.7	1.2	1.7
Estimated *K* (×10^4^ J/m^3^)	1.015	1.693	1.236	1.710
Real *M_S_* (Am^2^/kg)	30	80	30	40
Estimated *M_S_* (Am^2^/kg)	31.419	78.811	29.815	41.042
Real MNPs mass (μg)	10	40	90	100
Estimated MNPs mass (μg)	9.99	39.99	89.97	100.01

**Table 2:** Estimation results of parameters and iron mass under measured data.

	Trial 1	Trial 2	Trial 3	Trial 4
Estimated *K* (×10^4^ J/m^3^)	1.640	1.631	1.646	1.612
Estimated *M_S_* (Am^2^/kg)	60.334	59.682	63.271	61.131
Real MNPs mass (μg)	170	160	150	140
Estimated MNPs mass (μg)	169.95	159.84	149.48	140.27

## Data Availability

The original contributions presented in this study are included in the article. Further inquiries can be directed to the corresponding author.
